# Values and Principles as Cornerstones of a Renewed Normal

**DOI:** 10.1007/978-3-030-65355-2_26

**Published:** 2021-03-20

**Authors:** Corien Prins

**Affiliations:** 1 Tilburg University, Tilburg, The Netherlands; 2 Tilburg University, Tilburg, The Netherlands; 3 Tilburg University, Tilburg, The Netherlands; 4 Tilburg University, Tilburg, The Netherlands; Tilburg Institute of Law, Technology and Society, Tilburg Law School, Tilburg, The Netherlands

## Abstract

The effects of the outbreak of the coronavirus on our health, our economy, and our societies are being seen and felt around the planet. In countless ways, our society and familiar customs are being disrupted and challenged. In this light, we frequently hear that we are simply going to have to learn to live with this “new normal” and a socially distanced economy. And certainly, as long as no vaccine or treatment is available, this seems inevitable. But once a vaccine or treatment is available: What are the changes that remain in place and to what extent will our society have reinvented itself?

The effects of the outbreak of the coronavirus on our health, our economy, and our societies are being seen and felt around the planet. In countless ways, our society and familiar customs are being disrupted and challenged. In this light, we frequently hear that we are simply going to have to learn to live with this “new normal” and a socially distanced economy. But given a vaccine now becomes available: What are the changes that remain in place and to what extent will our society have reinvented itself?

## To Roll Back Crisis Solutions

Aside from the fact that, once the vaccine is broadly available, some of today’s emergency measures will no longer be necessary, we should also acknowledge that certain measures cannot remain in place indefinitely, among others, due to their invasiveness. For example, digital technology has proven to be a powerful tool in both tackling the spread of the coronavirus and allowing countless societal processes to continue. However, some of its current applications are at odds with the principles of the law and with fundamental rights. Consider, for instance, concerns regarding the protection of privacy in relation to digital monitoring of citizens’ behavior (such as tracking apps; the use of data about restaurant reservations for coronavirus monitoring, etc.). But equally, the benefits of some of these new digital ways of working (greater efficiency, reduced costs, etc.) should not be allowed to lead, insidiously, to a society in which certain groups in our society are (even more) disadvantaged in socio-economic terms. After all, a good laptop and internet connection are not available to every family. And not everyone is able to keep pace with the sudden digital transformation of so many key societal processes. Similarly, there are concerns regarding the continued focus on the use of digital learning environments in higher education. The current crisis has meant that our education system has become even more dependent on a few very large tech companies (mainly based in the US). In providing their services, these commercial companies are able to collect user data from students and researchers, which can be used to offer personalized marketing and services. In essence, digitalization in higher education increasingly also means the privatization of tasks that have traditionally belonged to the public domain. What does the continuing digitalization of education mean for the public values that our system of education is meant to serve? Now that we are taking careful steps towards a relaxation of the measures, it is a good time to discuss the desirable intensity of the use of digital applications. Everything had to be done in a hurry in the initial phase. In the period ahead, there will be more time for reflection, and the question lies before us: what kind of balance between physical and digital—between offline and online—are we looking for? Digitalization has a lot to offer, but at the same time, it can change or diminish things fundamentally to such an extent that we need to ask ourselves if those changes are indeed desirable. A debate is vital, particularly now it has become clear that some changes must not be embraced permanently and even come with the necessary risks attached (Sheikh and Prins 2020).

The need for a public debate on the responsible use of digital technology and the relevant values associated with that is just one illustration of the fact that the current crisis and its aftermath are pushing many new challenges and questions onto the agenda. In this short contribution, I would like to frame two such questions in particular. From a legal point of view, what would the equivalent be of the socially distanced economy? And secondly, from the point of view of the law and the rule of law, which considerations do we need to focus on if we are to reinvent our society?

## Socially Distanced: A Metaphor for a Renewed Legal System

The initial response to the first question—the equivalent of the socially distanced economy—probably centers on the numerous legal provisions that have been affected by the crisis and, in addition, the provisions that have been implemented to cope with the (effects of the) crisis. These include things like emergency decrees, rules regarding economic support measures, the relaxation of certain rules in the field of competition or healthcare, and the interpretation of certain notions in contract law, such as that of unforeseen circumstances. It has been necessary to review these aspects of the law in order to soften the impact of the crisis or to share the pain more equitably or because they were preventing action being taken to manage the situation effectively. At the same time, of course, some new legal measures have been introduced too, including rules to prevent unscrupulous individuals from profiting from others’ misfortunes by charging excessive prices for certain products.^1^

However, hopefully, the legal equivalent of social distancing means more than just a few technical changes to the application of the law or the more flexible application of existing legislation and legal concepts. After all, social distancing is essentially all about protecting the most vulnerable in our society and ensuring solidarity. This means that we need to look at the architecture of the law from a new perspective. This implies that we aim to take it one step further than merely the legal measures that we take at this very moment to deal with the crisis. After all, does the fact that countless aspects of the law have had to be overridden or amended not make it painfully clear that our legal system is inadequately equipped to support or sometimes even prioritize the most vulnerable? Does it not show that the weakest and most vulnerable in our society have been neglected and forgotten to some extent when it comes to the law? There are plenty of examples of this in my field of expertise—law and technology. At the global level, for example, extremely restricted use is made of the compulsory license in patent law even though two billion people in the world still have no access to affordable basic medication (Durisch and Gajardo 2018). And, incidentally, this does not only happen in the African continent but in the Western world too. For example, the political prioritization of international trade treaties has meant that a country like Canada, which had actively been using compulsory licenses to import generic medicines, was obliged to abandon the use of this instrument. The prices of medicines in that country rose sharply as a result (‘t Hoen 2009).

This is just one example of what can be seen as a much more generalized trend. Years of beating the drum for “self-reliance” and “competition” have also affected the structures and reflexes of our system of law. The discipline of the market and the dominance of each individual’s responsibility for his/her own life (and also, therefore, for any misfortune that comes his/her way in life)—all combined with an emphasis on the most efficient possible use of capacity, capital, and technology—have colored the way in which we apply the law as well as the law itself. Although almost every field of the law has provisions in place to ensure that the public interest cannot be ignored completely, the legal provisions that serve to underscore public values and embody fundamental norms often belong in the category of “exceptions” to the general provisions of the law, rather than fundamental principles.

## Values and Principles Underlying the Renewed Normal

A crisis such as the one we are currently witnessing means that, in many areas of society, we are forced to rethink established patterns and familiar arrangements. This includes the arrangements that we, as a society, have made in the form of the law. The results of this rethink will become tangible in the months and years to come in the form of numerous new or amended laws, court rulings, and agreements between parties. Various areas of law will be redesigned, ranging from social security for self-employed persons to better control over the supply of medical equipment.

But let us, above all, make sure that the legacy of this crisis goes beyond just these areas. The coronavirus is forcing us to confront our own vulnerability as human beings. And this ought to make us reconsider the way in which we distribute or redistribute wealth and promote solidarity within our societies: pensions, insurance, housing, health care, and education must be the main areas that we focus on. After all, this is how we create a strong middle class on which every robust democracy is built. But the new panorama that is opening up before us must also extend to countless “minor” arrangements that our legal system currently provides for, from civil liability to special tax arrangements for multinational companies. Above all, let us make sure that social distancing serves as a reminder of the role of the law in promoting solidarity with other people and the acceptance of measures that are designed primarily in the interests of other people.

Sustainable change will require an appreciation of both new and traditional ways of working and living together and the ways in which we interact socially and economically. The sum of all these changes will result in what could be referred to as “the renewed normal.” For it is important to realize that, in this new reality, certain principles and underlying values in our democratic society will need to be preserved as well. In other words, emergency solutions and their short-term benefits (increased efficiency, reduced costs, etc.) should not be allowed to lead (insidiously) to practices that are actually at odds with the principles on which our society is based. Hence, it is crucial that we reflect on the values in our democracy that must be saved from being washed away in the tide of the current crisis.

This brings me to summarize and formulate a crucial issue for the agenda of academic and political thought and discussion. Clearly the above shows that the numerous challenges we face in light of the coronavirus crisis definitely includes the challenge of reflecting on which values and principles our reinvented society and renewed normal should be based.

## Footnotes

1 For example, the new legal regime in Ontario: https://www.ontario.ca/laws/regulation/200098.
